# *Cryptococcus neoformans* endocarditis in an immunocompetentpatient a case report

**DOI:** 10.1186/s12872-022-02997-9

**Published:** 2022-12-23

**Authors:** Colin N. McGuire, Dylan J. Walter, Samuel B. Wopperer

**Affiliations:** Department of Internal Medicine, MedStar Georgetown University Hospital, 3800 Reservoir Rd NW, Washington, DC 20007 USA

**Keywords:** Cryptococcal endocarditis, Cryptococcal fungemia cardiac manifestations, Cryptococcal infection in heart failure, *Cryptococcus neoformans* valvular disease

## Abstract

**Background:**

*Cryptococcus neoformans* is an invasive fungal infection commonly affecting immunocompromised patients as pneumonia or meningitis. More rarely, case reports describe *Cryptococcus neoformans* endocarditis, though nearly exclusively among patients with active immunosuppression, implanted cardiac devices or prosthetic valves.

**Case presentation:**

We report the case of a patient with underlying substance abuse disorder and systolic heart failure presenting with fever, altered mental status, and shower emboli subsequently found to have native tricuspid valve vegetations and blood cultures positive for *cryptococcus neoformans* in the absence of immunosuppression.

**Conclusions:**

Historically, *Cryptococcus neoformans* fungemia manifests clinically as pneumonia or meningitis among the immunosuppressed. There have been rare reports of endocarditis in this population and even fewer reports of native valve endocarditis exist. The present case along with mortality reported in prior literature, suggest suspicion must be maintained in the absence of immunosuppression, even in patients with native valves.

## Highlights

- Cryptococcal Endocarditis poses risk in non-immunosuppressed patients
- Aortic and Mitral Valve most affect by Cryptococcus
- Mortality remains high in Cryptococcal Endocarditis
- Tabulated review of available Cryptococcal Endocarditis literature

## Background

*Cryptococcus neoformans* (CN) is an encapsulated yeast found throughout the world known to cause nearly half a million deaths per year. Immunocompromised patients are predominantly affected [1, 2]. Most commonly, CN presents as pneumonia or meningitis though rare cases of infective endocarditis are described [3, 4]. Current literature posits a majority of the CN endocarditis in non-immunosuppressed patients in association to implanted cardiac devices and prosthetic valves [5]. Herein, we describe the novel presentation and clinical course in an immunocompetent patient with native heart valves.

## Case presentation

A 65-year-old female with a past medical history significant for heart failure with reduced left ventricular ejection fraction, chronic obstructive pulmonary disease, stage III chronic kidney disease and substance abuse disorder presented with altered mental status. On presentation, she had notable bilateral conjunctival hemorrhages, splinter hemorrhages, and signs of hypervolemia to include jugular venous distension and pitting edema of both lower extremities. “Track marks” were evident in the cubital fossa along with scattered hyperpigmented ovaloid lesions consistent with “skin popping” injuries. Admission laboratory results showed elevated blood urea nitrogen 65 mg/dL, hyperkalemia of 6 mmol/L, acute kidney injury (creatinine of 4.93 mg/dL from baseline 1.57 mg/dL) and a urinalysis consistent with urinary tract infection, white blood cell count showed no leukocytosis (WBC 5.6 k/uL), hemoglobin showed anemia (Hgb 10.8 gm/dL), platelets were 172 k/uL, a coagulopathy with prothrombin 16.7 s, international normalized ratio 1.9, liver function showed aspartate aminotransferase of 48 units/L, alanine transaminase 21 units/L, and elevated high sensitivity troponin I 838 ng/L which decreased over the following hospital days. Further, the patient was COVID-19 positive. Dexamethasone, remdesivir, and aztreonam were given at initial presentation. Her urine cultured positive for *Proteus mirabilis* (10,000 to 100,000 cfu) and antibiotics were narrowed to reported sensitivity on hospital day 2. Nonetheless, she persistently reported chills and demonstrated continued alteration of mental status. Blood cultures were negative.

**Fig. 1:**
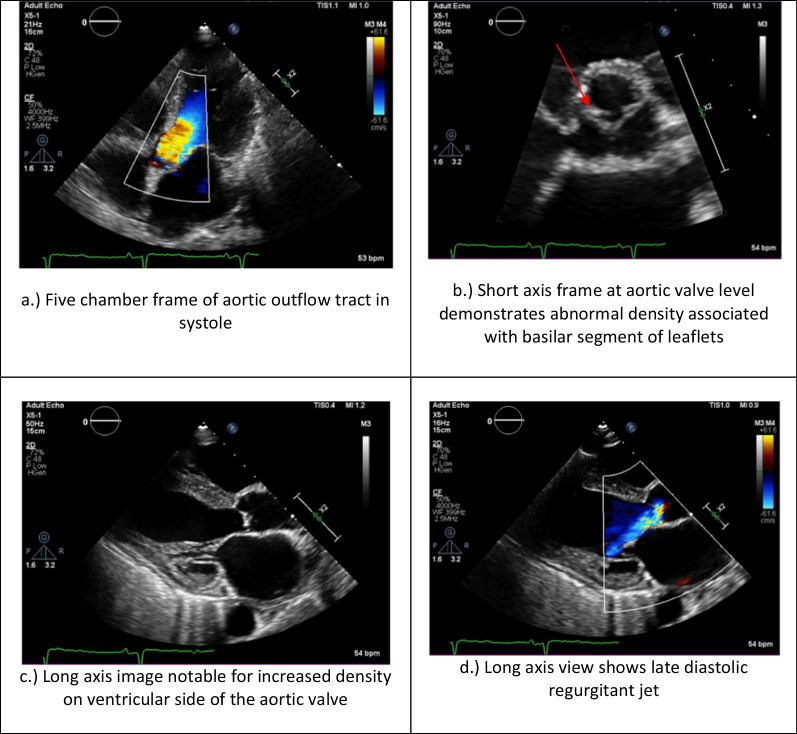
Transthoracic echocardiogram series

**Fig. 2:**
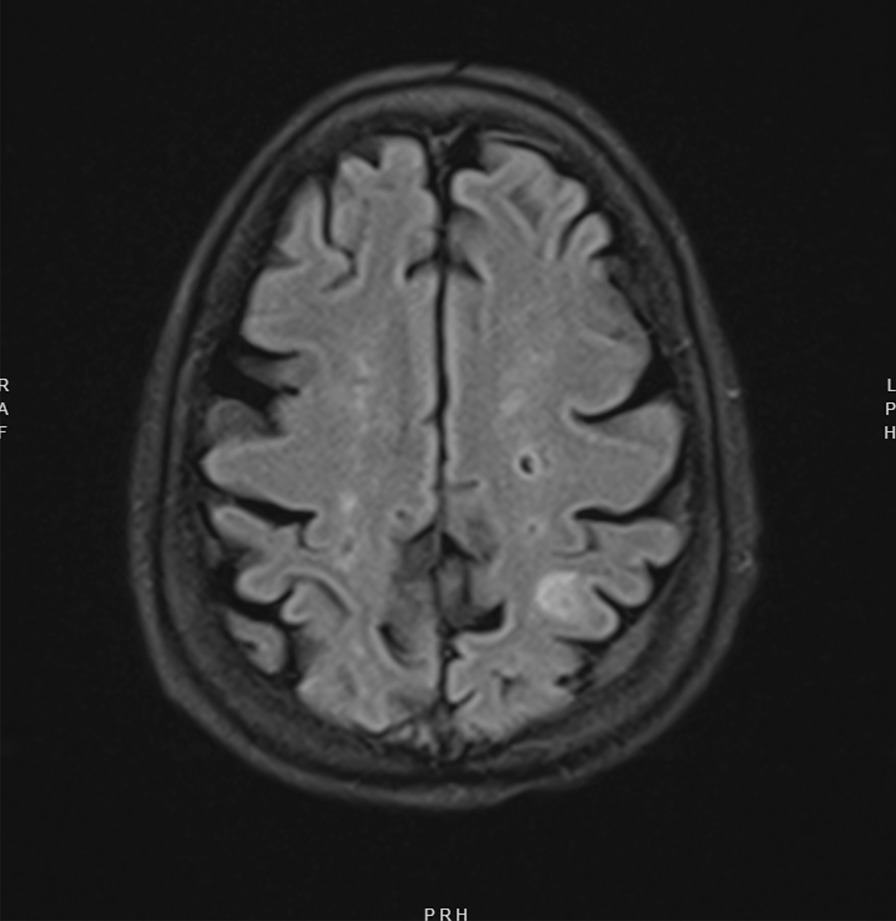
MRI Brain demonstrates prominent new left sided frontal and parietal foci consistent with small acute infarction and septic emboli

**Fig. 3:**
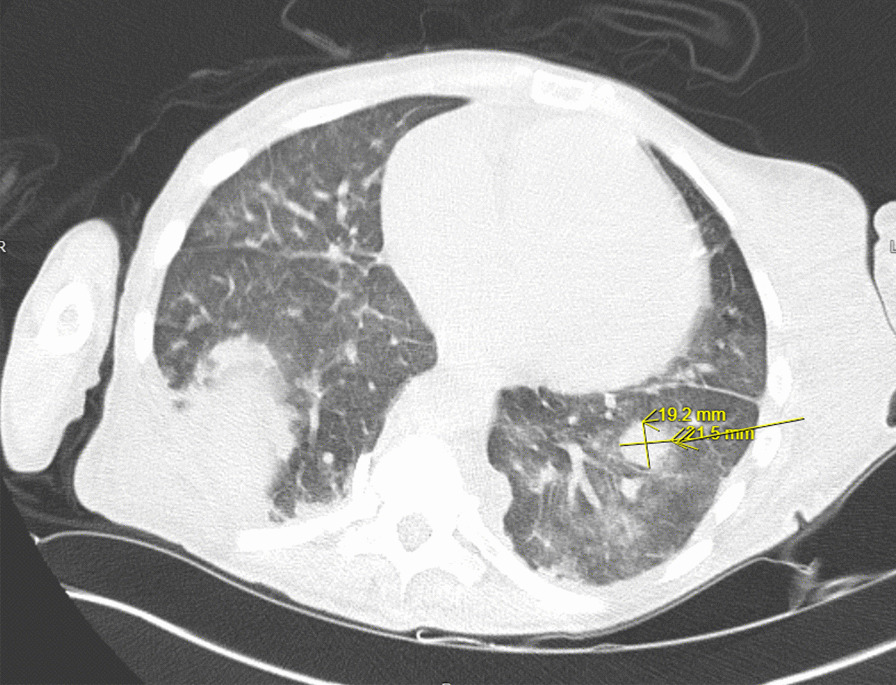
CT chest, abdomen, pelvis with coalescing regions of opacities and nodular density within the left lower lobe

**Fig. 4:**
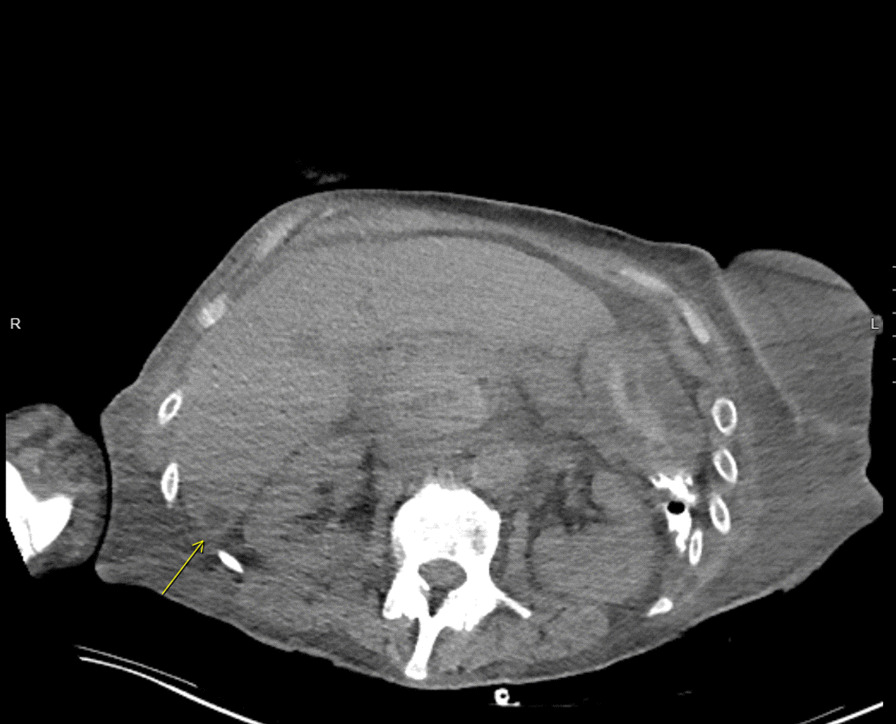
CT chest, abdomen, pelvis indicating the hypoattenuating liver nodule

A transthoracic echocardiogram completed on hospital day 3 for her volume overload and acute decompensated heart failure highlighted interval worsening of her moderate-severe aortic regurgitation and new small mobile echo densities on the ventricular side of the aortic valve, suspicious for vegetation (Fig. 1). Computed tomography of the head on hospital day 3 revealed several foci of hypodensity in the frontal and parietal lobes, confirmed by immediate follow-up MRI to indicate acute infarcts (Fig. 2). Concern for other embolic phenomena prompted CT of the chest, abdomen, and pelvis which returned positive for new nodular density in the left lower lung lobe (Fig. 3) and scattered hypoattenuating nodules in the liver (Fig. 4). An abnormal signal in the pancreatic head not amenable to characterization beyond mass versus embolic sequalae was also noted. Heparin infusion was initiated given a concern for diffuse emboli. A week after admission, blood culture resulted positive for cryptococcus. Anti-fungal coverage with micafungin and flucytosine was initiated immediately. On hospital day 11 the patient developed thrombocytopenia (platelet count 30 k/uL) and heparin infusion was ceased for possible heparin induced thrombocytopenia.

Positive Duke’s criteria in the patient included (1) evidence of endocardial involvement on echocardiogram, (2) predisposing risk factor (IVD’s), (3) presence of vascular phenomenon (conjunctival and splinter hemorrhages, septic pulmonary infarct, cerebral emboli) and (4) blood culture positivity. Serum cryptococcal antigen titers returned 1:256 on hospital day 12. After positive antigen titers, Amphotericin was added to flucytosine. On hospital day 13, lumbar puncture revealed an opening pressure of 26 mmHg, glucose 71 mg/dL, protein 32 mg/dL, and lymphocytes 79 microL. Cerebrospinal fluid culture was completed and negative as were Human immunodeficiency virus (HIV) and immunoglobulin panels. Transesophageal echocardiogram was planned for hospital day 17 but the patients worsening mixed septic and cardiogenic shock resulted in respiratory and hemodynamic failure causing death.

## Discussion

Rates of fungal endocarditis rate are rising and are estimated to comprise 1–10% of all infective endocarditis with a total of 1.5 to 11.6 cases per 100,000 [6]. The most common fungal infection is *Candida albicans* (24–46%), followed by *Aspergillus* (25%) [7]. Fungal endocarditis is typically a progression from initial fungemia when the cardiac valves are seeded. A prominent risk factor among non-immunocompromised hosts is IV-drug use [8].

More frequently, CN infections affect immunocompromised patients. Incidence is 2–7 per 1000 among HIV positive patients in the United States and confers substantial mortality risk at a rate of 12% [9]. Epidemiology of non-immunocompromised hosts and the underlying infection rates of CN is elusive and less clear [10]. The most common underlying risk factors have been described as diabetes mellitus, end-stage renal disease, liver cirrhosis, and IV-drug use [8, 10]. Non-immunocompromised patients have proportionately less CNS involvement and cryptococcemia compared to immunocompromised patients [10].

CN heart disease is previously reported as presentation of mycotic endocarditis, pericarditis, and cardiomyopathy [5, 11, 12]. Many prior cases have involved implantable prosthetic cardiac devices (prosthetic valves or AICD’s). Patients without implanted devices are frequently immunosuppressed. Only 2 prior cases describe patients void of active immunosuppression and implanted cardiac devices. Among these, IVD use is a unifying risk factor [4, 5, 13–21].Diagnosis of fungal endocarditis historically hinges on the Duke Criteria which may preclude a diagnosis if the infection is caused by nontypical microorganisms. In this vain, it remains imperative for the clinician to maintain high index of suspicion in patients with risk factors [22]. In cases of CN endocarditis, a majority of blood cultures will return positive (Table 1).

**Table 1:** Literature review of published cases with *Cryptococcous neoformans* endocarditis

Reference	Year	Age, sex	Symptoms	Comorbidities	ISx	Serum testing	Diagnostic imaging	Infectious focus	Valve location	Treatment	Complication	Survival
Harford et al. [14]	1974	51, M	Fever	Pancytopenia	+	Blood Cx	Autopsy	Prosthetic	AV	Amphotericin B	Pneumonia, Abdominal wall abscess	0
Boden et al. [16]	1979	56, M	Weakness, Delirium	Hematologic malignancy	+	Cryptococcal antigen	TTE	Native	MV	Amphotericin B	Meningitis, MI	0
Blanc et al. [17]	1983	27, M	Fever, Seizures	CKD, AR	+	Blood Cx	Surgery	Prosthetic	AV	Amphotericin B, Flucytosine	Meningitis, Cardiac abscess	+
Banerjee et al. [18]	1996	12, M	Fever	Rheumatic heart disease	0	Blood Cx	TTE	Prosthetic	MV	Amphotericin B, Fluconazole	Cerebral abscess	+
	2020	57, F	Headache, Fatigue, Syncope	Cardiomyopathy, AICD Implantation, Vasculitis	+	Blood Cx	TTE, TEE	AICD Lead	0	Amphotericin B, Flucytosine, Fluconazole 6 weeks	Meningitis	+
Jone et al. [12]	1997	48, M	Fever, Surgical dehiscence	Rheumatic heart disease	0	Thrombus Cx	None	Prosthetic	0	Amphotericin B	Cerebral infarction	0
Alhaji et al. [5]	2011	41, M	Fever, Weight loss, Cough, Dizziness	CKD, Bicuspid aortic valve	0	Blood Cx	TEE	Prosthetic	AV	Amphotericin B	0	+
Sajjadi et al. [6]	2018	26, M	Shortness of breath	IV-drug use	0	Blood Cx	TTE	Native	AV	Amphotericin B, Fluconazole	0	+
Child et al. [15]	2018	46, M	Hemiplegia	Leukemia	+	Vegetation Cx	TTE	Native	MV	Voriconazole, Capsofungin	Cerebral infarction	+
Nakajima et al. [4]	2019	72, M	Fever, Respiratory failure	Diabetes, Interstitial pneumonia, AICD	+	Blood Cx	TEE	AICD Lead	0	Micafungin, Fluconazole, Amphotericin B	Meningitis	0
Kowatari et al. [19]	2020	50, M	Fever, chest pain, shortness of breath	CVA, Hepatitis B	0	Blood Cx	TTE	Native	AV	Surgery, Cefminox, Amphotericin B, Fluconazole 10 weeks	0	+
Present case	2021	65, F	Shock, Bradycardia, Delirium, Respiratory failure	COVID-19 pneumonia, CKD, COPD, IV-drug use	0	Blood Cx	TTE	Native	AV	Amphotericin B, Flucytosine, Fluconazole	Cerebral infarction, Meningitis, Cardiogenic shock	0

Case Summaries to Date: Summary table of all case reports involving *Cryptococcus neoformans* endocarditis. 12 documented cases in literature from 1957 to 2021. Ages range 4 years to 65 years old. 10 males, 2 females. Common symptoms at presentation include fever, pulmonary complaints, and delirium. The most common comorbidities include chronic kidney disease, immunosuppression, valvular disease and intravenous drug use. Immunosuppressed patients are more often infected. Non-suppressed patients frequently had prosthetic heart valves. Only the aortic and mitral valves have been implicated. 3 cases of immune-competent patients without a prosthetic valve are known. Treatment has included amphotericin B. in every case. Medical complications commonly include meningitis and cerebral infarction. Death occurred in 5 out of 12 cases

*AICD* automated implanted cardiac defibrillator, *AR* aortic regurgitation, *AV* aortic valve, *CKD* chronic kidney disease, *COPD* chronic obstructive pulmonary disease, *CVA* cerebral vascular accident, *F* female, *ISx* immunosuppression, *IV*: intravenous, *M* male, *MI* myocardial infarction, *MV* mitral valve, *TEE* transesophageal echocardiogram, *TTE* transthoracic echocardiogram, + present/yest; 0: absent/no.

Table 1 summarizes characteristics in the previously available literature CN endocarditis cases. Patient age ranges 4 years to 65 years old. 10 males and 2 females have been affected. Common symptoms at presentation include fever, pulmonary complaints, and delirium. The most common comorbidities include chronic kidney disease, immunosuppression, valvular disease and intravenous drug use. Immunosuppressed patients are more often infected. Non-suppressed patients frequently had prosthetic heart valves. To date, only the aortic and mitral valves have been implicated. 3 cases of immune-competent patients without a prosthetic valve are known. Treatment has included amphotericin B. in every case. Common medical complications include meningitis and cerebral infarction. Death occurred in 5 out of 12 cases.

Neurologic complications of Cryptococcus n. endocarditis are common, often manifesting as fungal meningitis, cerebral infarctions, or septic emboli (Table 1). Lumbar puncture should be performed to exclude CNS infiltration. In bacterial infective endocarditis, acute ischemic strokes are the most common sequalae, noted in 30–40% of cases. Cerebral infarction (27%), meningitis (1–20%), septic emboli (2–4%) and abscess (1–7%) are other frequent complications. Paucity of literature exists detailing incidence of neurologic involvement in patients with fungal endocarditis [22], though we have documented septic emboli, strokes, and meningitis in our present review.

## Data Availability

All data generated or analyzed during this study are included in this published article [and its supplementary information files].

## Abbreviations

CN: *Cryptococcus neoformans*
HIV: Human immunodeficiency virus
