# Correction to: *Cryptococcus neoformans* endocarditis in an immunocompetentpatient a case report

**DOI:** 10.1186/s12872-023-03170-6

**Published:** 2023-04-17

**Authors:** Colin N. McGuire, Dylan Walter

**Affiliations:** Department of Internal Medicine, MedStar Georgetown University Hospital, Washington, DC USA

**Correction to: BMC Cardiovascular Disorders (2022) 22:565**

10.1186/s12872-022-02997-9

Following publication of the original article [1], the author name “Samuel B. Wopperer” is deleted from the author group.

The original article has been corrected.

Author details: 1Department of Internal Medicine, MedStar Georgetown University Hospital, 3800 Reservoir Rd NW, Washington, DC 20007, USA
